# Engineering the Dimensional Interface of BiVO_4_-2D Reduced Graphene Oxide (RGO) Nanocomposite for Enhanced Visible Light Photocatalytic Performance

**DOI:** 10.3390/nano9060907

**Published:** 2019-06-10

**Authors:** Jing Sun, Chunxiao Wang, Tingting Shen, Hongchen Song, Danqi Li, Rusong Zhao, Xikui Wang

**Affiliations:** 1 School of Environmental Science and Engineering, Qilu University of Technology (Shandong Academy of Sciences), Ji’nan 250353, China; wangyiwangchunxiao@163.com (C.W.); stthunanyt@163.com (T.S.); shc01261005lx@gmail.com (H.S.); autantenporte@163.com (D.L.); 2 Key Laboratory for Applied Technology of Sophisticated Analytical Instruments of Shandong Province, Analysis and Test Center, Qilu University of Technology (Shandong Academy of Sciences), Ji’nan 250014, China; zhaors1976@126.com; 3 College of Environmental Science and Engineering, Shandong Agriculture and Engineering University, Ji’nan 251100, China

**Keywords:** BiVO_4_, RGO, visible light, two-dimensional interface, photocatalysis

## Abstract

Graphene as a two-dimensional (2D) nanoplatform is beneficial for assembling a 2D heterojunction photocatalytic system to promote electron transfer in semiconductor composites. Here a BiVO_4_ nanosheets/reduced graphene oxide (RGO) based 2D-2D heterojunction photocatalytic system as well as 0D-2D BiVO_4_ nanoparticles/RGO and 1D-2D BiVO_4_ nanotubes/RGO nanocomposites are fabricated by a feasible solvothermal process. During the synthesis; the growth of BiVO_4_ and the intimate interfacial contact between BiVO_4_ and RGO occur simultaneously. Compared to 0D-2D and 1D-2D heterojunctions, the resulting 2D-2D BiVO_4_ nanosheets/RGO composites yield superior chemical coupling; leading to exhibit higher photocatalytic activity toward the degradation of acetaminophen under visible light irradiation. Photoluminescence (PL) and photocurrent experiments revealed that the apparent electron transfer rate in 2D-2D BiVO_4_ nanosheets/RGO composites is faster than that in 0D-2D BiVO_4_ nanoparticles/RGO composites. The experimental findings presented here clearly demonstrate that the 2D-2D heterojunction interface can highlight the optoelectronic coupling between nanomaterials and promote the electron–hole separation. This study will motivate new developments in dimensionality factors on designing the heterojunction photocatalysts and promote their photodegradation photocatalytic application in environmental issues.

## 1. Introduction

Semiconducting nanocrystals with tailored shapes have attracted increasing research attention in recent years due to their many intrinsic shape-dependent properties [1,2]. As an important ternary oxide semiconductor, BiVO_4_ has been extensively investigated due to its peculiar chemical and physical functions in many fields such as dye-treatment, oxygen production, antibiotics degradation and so on [3,4,5]. However, the specific surface area of BiVO_4_ is comparatively small mainly due to the large particle size [6]. The poor adsorptive performance and the poor separation efficiency of photoinduced charge carriers in pure BiVO_4_ significantly restricts its further photocatalytic application [7].

To improve the photocatalytic performance of BiVO_4_ photocatalysts, many approaches have been explored, such as combining with metal oxides, doping metal ions and nano-structuring [8,9,10]. In particular, the photocatalyst hybrids with heterojunction systems represent an effective way to enhance the photoinduced electron and holes separation. The build-in internal electric field caused by the interface of hybrids promotes the electron flow across the heterojunction [11]. Generally speaking, the electron transfer (ET) across the heterojunction interface is a key process in controlling their photocatalytic performance [12]. The challenge of assembling the heterojunction systems lies in finding an appropriate platform favorable to electron transfer between the interfaces.

Among various materials, graphene, two dimensional forms of sp^2^-hybridized carbon, has exhibited outstanding characteristics such as high mechanical strength, thermal and optical properties and high electrical conductivity [13,14,15]. It can offer new opportunities to serve as an ideal platform to assemble the heterojunction systems. Research works have reported that the low-dimensional heterojunctions based on graphene is proven effective for ET process. For example, graphene combined with BiVO_4_ nanoparticles or nanotubes has been synthesized and exhibits high visible-light-driven catalytic effect [7,16]. Recently, 2D dimensional heterojunctions with superior properties have motivated considerable interest in degrading pollutants. Inspired by the process for light-charge conversion in granum of green plants, 2D-2D dimensional heterojunction with BiVO_4_ nanosheets-graphene stacked structures was fabricated to achieve rapid charge transfer [17,18]. Graphene, as an ideal 2D platform for photocatalysts assembly, benefits the electron transfer across the interface.

Recently, many literatures about BiVO_4_ and RGO composites have been reported. However, a thoughtful and systematic comparison in BiVO_4_-graphene nanocomposites with different dimensional heterojunctions is still scarce. Although 2D-2D dimensional heterojunctions with BiVO_4_/graphene exhibit superior photocatalytic performance, different preparation methods make the materials incomparable. The contribution role of different BiVO_4_ nanomaterials to enhance the composites photocatalytic activity is still unavailable. The situation may give incomplete or exaggerate information on the contribution of 2D-2D dimensional heterojunction in improving the photocatalytic performance [19]. So far, our knowledge of the specific advantages of 2D interface on developing an effective photocatalytic system is far from satisfactory.

Here a BiVO_4_ nanosheets/RGO based 2D-2D heterojunction photocatalytic system as well as 0D-2D BiVO_4_ nanoparticles/RGO and 1D-2D BiVO_4_ nanotubes/RGO nanocomposites were constructed by a feasible solvothermal method. Systematic comparison with the above nanocomposites was carried out in terms of photocatalytic activity, reactive oxygen species (ROS) generation and electron transfer rate. The results emphasize the key role of interfacial dimensionality on design or fabricate graphene-semiconductor nanocomposites and improvement of the photocatalytic activity.

## 2. Materials and Methods 

### 2.1. Materials

Following reagents were used: BiCl_3_, ethanolamine and acetaminophen (Aladdin Biochemical Technology Co., Ltd., Shanghai, China), Graphite powder and NH_4_VO_3_ (Bodi Chemical Co., Ltd., Tianjin, China), Bi(NO_3_)_3_·5H_2_O and ethylene diamine tetraacetic acid disodium salt (EDTA-2Na) (Tianjin Damao Chemical Reagent Co. Inc., Tianjin, China), formic acid, isopropanol (IPA) and p-Benzoquinone (Sinopharm Chemical Reagents Co., Ltd., Shanghai, China). All chemicals were used as received without further purification. Deionized water was used throughout the experiment.

### 2.2. Synthesis of BiVO_4_/RGO Composites

Graphene oxide (GO) was prepared using a modified Hummers’ method published in our previous work [20]. BiVO_4_ nanosheets/RGO composites and BiVO_4_ nanotubes/RGO composites were synthesized by the hydrothermal method, modified from previously reports [21]. Typically, 158 mg of BiCl_3_ powder and 59 mg of NH_4_VO_3_ powder were added in 50 mL deionized water and stirred for 30 min to produce a homogenous suspension. Then, certain amount of 1 M ethanolamine (0.3 mL for BiVO_4_ nanosheets; 2.5 mL for nanotubes) was added dropwise. After that, 6.32 mL 1 g L^−1^ GO solution was gradually added into the solution and then sonicated for 30 min to make the mixture uniform. The above solution was poured into a 100 mL Teflon-lined autoclave and reacted at 160 °C for 12 h. After cooling to room temperature, the resulting yellow precipitates in the reactor are collected and washed several times with alcohol and deionized water. Finally, the as prepared catalysts were dried at 60 °C for several hours. BiVO_4_ nanosheets and BiVO_4_ nanotubes were synthesized by a similar method without GO addition.

BiVO_4_ nanoparticles was prepared by a modified method according to the literature [22]. The BiVO_4_ nanoparticles/RGO composites were prepared by a hydrothermal method. The details are described in the Supplementary Materials.

### 2.3. Characterization

The powder X-ray diffraction (XRD) analysis was measured by Bruker-axs D-8 advance diffractometer (Cheshire, UK) with Cu Kα radiation. The morphology and element composition were recorded by using Scanning electron microscope (FE-SEM, Hitachi Regulus 8220, Tokyo, Japan). Raman measurements were acquired on a Bruker Senterra R200-L Raman spectrometer (Ettlingen, Germany). The optical adsorption behavior of the samples was performed on a Cary 5000 UV-vis-NIR spectrophotometer (Agilient Technologies, SantaClara, CA, USA). The absorption spectra were obtained by analyzing the reflectance measurement with Kubelka-Munk (KM) emission function, *F(R∞)*. Optical band gap energy (*E*g) can be determined from the plot between *E* = 1240/λ_Absorp.Edge_ and [*F(R∞)hυ*]^1/2^ where *E* is the photonic energy in eV and *hυ* is the energy of an incident photon. X-ray photoelectron spectroscopy (XPS) analysis was analyzed by a Kratos Axis Ultra DLD (Manchester, UK) with Al Kα X-ray source (1486.6 eV). A Fluorescence Spectrophotometer (JASCO FP-6500, Tokyo, Japan) was used for photoluminescence (PL) measurement at the excitation wavelength of 420 nm.

### 2.4. Photocatalytic Experiment

Acetaminophen were used as the target degradation contaminants to evaluate the photocataytic activity of the prepared catalysts. Before illumination, the solution containing 150 mL of 10 mg L^−1^ acetaminophen and 0.15 g photocatalysts was sonicated and stirred for 30 min in dark to ensure an adsorption/desorption equilibrium. Then the above suspension was irradiated by 300 W Xe arc lamp (PLS-SXE 300, Perfectlight Co. Ltd, Beijing, China) with a UV-cutoff filter (λ > 400 nm). At a given time interval of irradiation, 2 mL aliquots were collected from the suspension and centrifuged. The residual concentration of organics in the aliquots was measured by a TU-1901 UV-vis spectrophotometer (Purkinje General Instrument Co., Ltd., Beijing, China). The concentration of acetaminophen was determined by high performance liquid chromatography (LC-20AT, Shimadzu, Kyoto, Japan) with an Agela Venusil MP C18 (0.46 μm × 250 mm) reverse-phase column equipped with UV-Vis detector (SPD-20A, Shimadzu, Kyoto, Japan) at 254 nm. The mobile phase was methanol: water (35:65, v/v) and the flow rate was 0.8 mL min^−1^. All the photocatalytic experiments were performed in triplicates and the mean values are reported. 

## 3. Results and Discussion

### 3.1. Characterization of the BiVO_4_/RGO Composites

**Figure 1:**
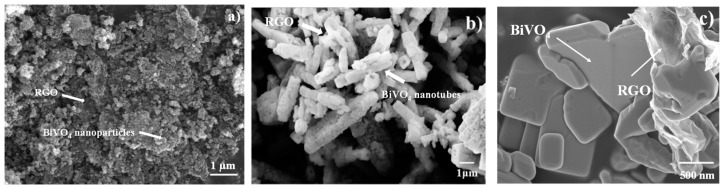
SEM images of BiVO_4_/ reduced graphene oxide (RGO): (**a**) BiVO_4_ nanoparticles/RGO; (**b**) BiVO_4_ nanotubes/RGO; (**c**) BiVO_4_ nanosheets/RGO.

BiVO_4_/RGO composites were synthesized employing covalent chemistry to achieve BiVO_4_ samples in situ growth on graphene surface. SEM and AFM images were taken to characterize the microscopic morphology and structure. Figure 1c and Figure S1 display the prepared BiVO_4_ nanosheet exhibiting two-dimensional sheet-like morphology with 500–1000 nm in width and 8–30 nm in thickness. The products layer on the RGO sheet platform, which forms the homogenous 2D-2D interfacial contact. Figure 1b indicated that the as-prepared BiVO_4_ nanotubes had a typical nanotubular structure and paved well on the RGO sheet to form 1D-2D heterostructures. During the synthesis, the formation of the 1D and 2D BiVO_4_ samples was controlled by the pH value of the solution. It is reported that an increase of the pH value will decelerate the nucleation kinetics and provide a more suitable condition for anisotropic growth of 2D or 1D m-BiVO_4_ nanostructures [21]. Similarly, the SEM images of BiVO_4_ nanoparticles/RGO (0D-2D) exhibit the well-dispersed BiVO_4_ nanoparticles on the RGO sheet in Figure 1a.

The RGO content of the above three composites are determined at ~2 wt% by TGA test in Figure S2. According to the literature, the amount of reduced graphene oxide was determined by the weight loss from 200 to 600 °C [23].

**Figure 2:**
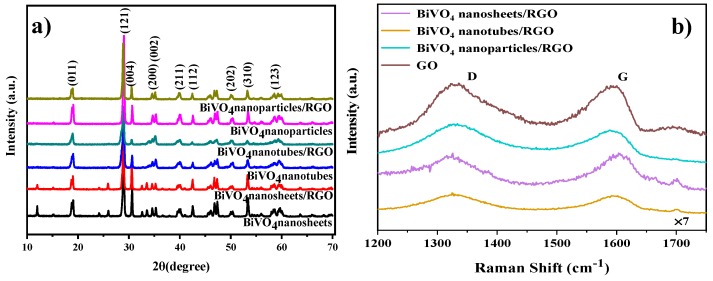
(**a**) XRD patterns BiVO_4_ and BiVO_4_/ RGO composites. (**b**) Raman spectra of graphene oxide (GO) and BiVO_4_/RGO composites in the 1200–1800 cm^−1^ region.

The phase and crystal structure of the as-prepared samples were examined by XRD. As shown in Figure 2a, the XRD patterns of BiVO_4_ and BiVO_4_/RGO samples all agree with the monoclinic scheelite type BiVO_4_ (JCPDS No.14-0688). Compared with BiVO_4_ nanoparticles and nanotubes samples, the dominant 004 diffraction peak suggests that BiVO_4_ nanosheets and BiVO_4_ nanosheets/RGO have a preferred orientation along the (001) planes [24]. Notably, for the sample GO of Figure S3, the peak at 2θ of 10.3° is attributed to the (002) reflection of stacked GO sheets. However, no diffraction peak of GO is observed in the composites, attributing to the disappearance of layer-stacking regularity after redox of graphite [8].

The monoclinic scheelite phase of BiVO_4_ in the composites is further confirmed by a typical Raman band at 126, 210, 325, 367, 710 and 828 cm^−1^ in Figure S4 [25]. In addition, the D band centered at 1350 cm^−1^ (disorder band) and the G band at 1580 cm^−1^ (tangential vibration band) are present, indicating that RGO has been successfully loaded on the surface of BiVO_4_ [26,27]. Furthermore, the *I_D_*/*I_G_* ratio is inversely proportional to the average size of the sp^2^-hybridized graphene domains [28]. As is shown in Figure 2b, after the solvothermal process, the *I_D_*/*I_G_* ratio of BiVO_4_ nanosheets/RGO decreased from 1.04 to 0.99, indicating that the reduction of GO increased the average size of the graphene domains and reduced the defect density in the composite [29]. Differently, an increase in the *I_D_*/*I_G_* ratio of BiVO_4_ nanoparticles/RGO and BiVO_4_ nanotubes/RGO are observed. The result shows that more numerous sp^2^ domains have been formed in the composites [28,30].

**Figure 3:**
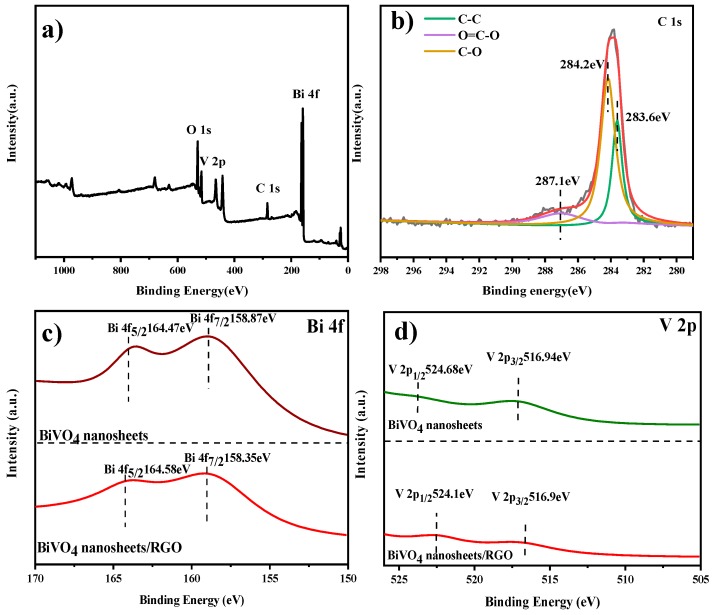
(**a**) The XPS survey spectrum of BiVO_4_ nanosheets/RGO and (**b**) C 1s band of BiVO_4_ nanosheets/RGO, (**c**) Bi 4f band of BiVO_4_ nanosheets and BiVO_4_ nanosheets/RGO. (**d**) V 2p band of BiVO_4_ nanosheets and BiVO_4_ nanosheets/RGO.

The chemical state of BiVO_4_ nanosheets and RGO in the composites is investigated by XPS in Figure 3. The survey spectrum in Figure 3a indicated the existence of Bi, V, O and C elements in BiVO_4_ nanosheets/RGO. In the C1s spectrum of BiVO_4_ nanosheets/RGO (Figure 3b), the peak centered at 283.6 eV binding energy indicates the existence of C–C bond from graphene. The peak located at 284.2 eV is attributed to the formation of C–O bond, suggesting the combination of BiVO_4_ nanosheets and RGO. The weak O=C–O bond centered at 287.1 eV indicates that the oxygenated functional groups of reduced graphene oxide are weakened during the hydrothermal reaction, along with the reduction of GO to RGO [31]. In Figure 3c, the two peaks at 158.87 and 164.47 eV are attributed to the orbital of Bi 4f_7/2_ and Bi 4f_5/2_, indicating the existence of Bi^3+^ in the pure BiVO_4_ nanosheets. However, in the BiVO_4_ nanosheets/RGO composites, the binding energy of Bi 4f_7/2_ is blue shifted to lower values by 0.5 eV, owing to the change of the chemical environment surrounding Bi element under the influence of RGO. The same results occurred in V 2p in Figure 3d. The observations in the XPS spectra further demonstrates the intense interaction between BiVO_4_ nanosheets and RGO.

**Figure 4:**
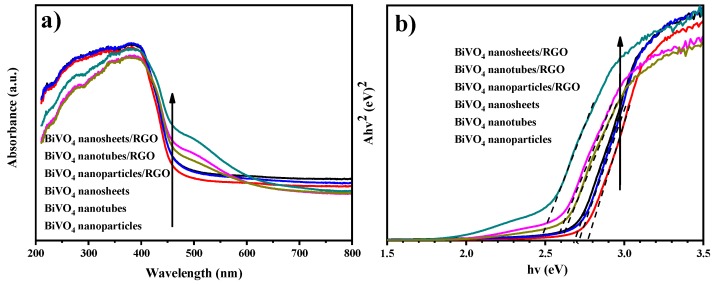
(**a**) UV-vis diffuse reflectance spectra of BiVO_4_ and BiVO_4_/RGO composites. (**b**) The relative band gap energy of the prepared samples.

The optical properties of the composites were analyzed by DRS. The UV-vis diffuse reflectance spectra can be used to determine the absorption edge information and the width of the forbidden band of catalysts. As shown in Figure 4a, the introduction of graphene enhanced the absorbance in the visible light region for the as-prepared BiVO_4_/RGO, especially for BiVO_4_ nanosheets/RGO. The optical band gap energy (*E*g) can be determined from the plot between *E* = 1240/λ_Absorp.Edge_ and [*F(R∞)hυ*]^1/2^ [32]. As shown in Figure 4b, the band gap energy of BiVO_4_ nanosheets/RGO is 2.45 eV, which is lower than that of pure BiVO_4_ nanosheets (0.21eV). These results demonstrate that the introduction of graphene in BiVO_4_ nanosheets/RGO nanocomposites can directly produce more excited charge transfer under visible-light irradiation, which is the premise of excellent photocatalytic performance.

### 3.2. Photocatalytic Performance of BiVO_4_/RGO Samples

As a common analgesic and antipyretic drug, acetaminophen is heavily used all over the world and detected in surface water, ground water and sewage effluents [33,34]. Once acetaminophen has overdosed, it may cause potential liver damage and even death [35,36]. Thus, it is particularly urgent to provide an efficient method to enhance the degradation of acetaminophen in wastewater. 

**Figure 5:**
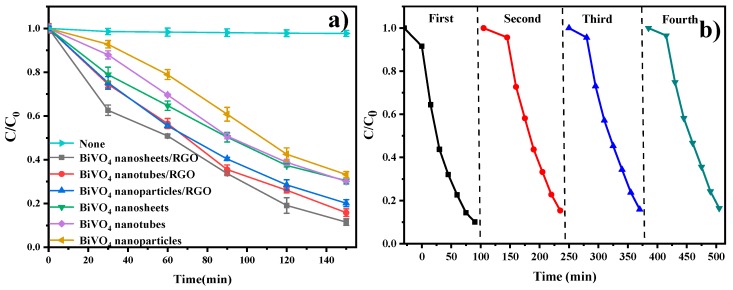
(**a**) Time-online photocatalytic performance of acetaminophen over the as prepared photocatalysts under visible light irradiation. (**b**) Stability experiments of BiVO_4_ nanosheets/RGO.

Hence, acetaminophen was used as a target pollutant to evaluate the photocatalytic properties of the prepared materials under visible light irradiation (λ > 400 nm). The chromatogram corresponding to the acetaminophen standard sample is shown in Figure S5 with the retention time at about 5.6 min. For comparison, BiVO_4_ nanoparticles (0D), BiVO_4_ nanotubes (1D), BiVO_4_ nanosheets (2D), BiVO_4_ nanoparticles/RGO (0D/2D), BiVO_4_ nanotubes/RGO (1D/2D), and BiVO_4_ nanosheets/RGO (2D/2D) were all examined. Figure S6 displayed the adsorption of acetaminophen on the nanomaterials reached an equilibrium state within 30 min in the dark. In Figure 5a, the photolysis performance of acetaminophen under visible light irradiation without any photocatalyst indicates that the self-degradation of acetaminophen is negligible under the visible light irradiation. The photodegradation curves of acetaminophen were fitted by pseudo-first-order reaction kinetics. As is shown in Figure 5a and Table S1, the addition of RGO can obviously improve the visible light performance of BiVO_4_ photocatalysts, indicating the heterojunction structure between BiVO_4_ and RGO contributed remarkably to the photocatalytic degradation rate. In particular, the BiVO_4_ nanosheets/RGO composites (*k* = 0.0141 min^−1^) exhibited the optimal performance compared with the corresponding pure BiVO_4_ (*k* = 0.0080 min^−1^). This demonstrates that the 2D-2D heterojunction structure is more beneficial to the photocatalytic activity than the other dimensional heterojunctions. When the 2D flake BiVO_4_ and the thin slice of RGO are parallel to the space, it is beneficial to maintain high photoelectron transport efficiency and reduce the recombination of the electron hole, improving the catalytic efficiency [37].

The amphoteric behaviour of the solution influences the surface charge of the photocatalyst [38]. The role of pH on the photodegradation efficiency was studied in the pH range 3–11. As is shown in Figure S7, the photodegradation efficiency of BiVO_4_/RGO samples increases with the increasing of pH and the maximum rate was at pH 11. That may be ascribed to major contribution of electrostatic interaction on mass transfer rate [39]. It is considered that under alkaline conditions, there is a large quantity of OH^−^ in the solution, which favours the formation of •OH. The strong oxidation of •OH plays an important role in the process of photocatalytic degradation [40]. Compared with BiVO_4_ nanotubes/RGO and BiVO_4_ nanoparticles/RGO, BiVO_4_ nanosheets/RGO showed more excellent catalytic performance under neutral conditions, indicating that the pH application range of flaky BiVO_4_/RGO was wider.

**Figure 6:**
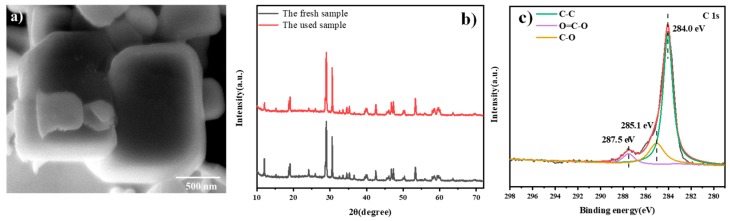
(**a**) SEM, (**b**) XRD and (**c**) XPS results of C 1s band of BiVO_4_ nanosheets/RGO after four cycles irradiation.

The stability of the BiVO_4_ nanosheets/RGO was evaluated by performing the recycling experiments. In Figure 5b, the photocatalysts can still maintain excellent degradation efficiency after four cycles. Figure 6a displays the smooth surface of BiVO_4_ nanosheet after visible light irradiation. Compared with the fresh sample, no obvious discrepancy in the XRD pattern of the recycled sample was observed in Figure 6b. As is shown in Figure 3b and Figure 6c, the XPS spectra of the recycled BiVO_4_ nanosheets/RGO exhibited a slight decrease of the C−O and O=C–O peak intensity, possibly attributed to the further photocatalytic reduction of GO to RGO during the photocatalytic degradation [20]. However, the above changes did not affect the photocatalytic performance after recycling experiments. It is proved that the BiVO_4_ nanosheets/RGO composites prepared exhibit relatively high stability.

### 3.3. Photocatalytic Mechanism of BiVO_4_/RGO

**Figure 7:**
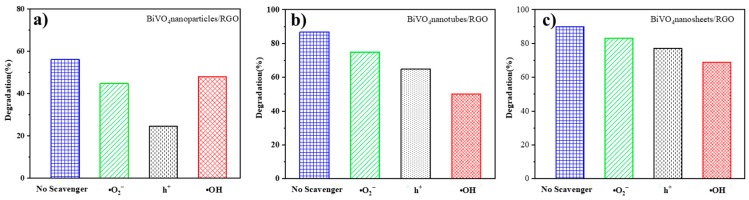
Free radical inhibition experiment of BiVO_4_/RGO: (**a**) BiVO_4_ nanoparticles/ RGO; (**b**) BiVO_4_ nanotubes/RGO; (**c**) BiVO_4_ nanosheets/RGO.

It is commonly accepted that a series of reactive species, such as hole (h^+^), hydroxyl radical (•OH), and superoxide radial (O_2_^•−^), usually govern the photocatalytic degradation reactions of organic pollutants [41]. In order to investigate the main reactive species responsible for the photocatalytic activity of BiVO_4_/RGO samples, the radical trapping experiment was carried out. Figure 7 displays the photocatalytic degradation curves of acetaminophen over BiVO_4_ nanotubes/RGO, BiVO_4_ nanoparticles/RGO, and BiVO_4_ nanosheets/RGO with the addition of ROS scavengers. In this experiment, isopropanol (IPA) was used to quench •OH, formic acid for h^+^, and p-benzoquinone (BQ) for O_2_^•−^. For comparison, the radical trapping results of the pure BiVO_4_ samples was demonstrated in Figure S8.

As depicted in Figure 7b,c, acetaminophen degradation process was obviously depressed by IPA, verifying •OH plays the most important role in the photocatalytic reaction over BiVO_4_ nanosheets/RGO and BiVO_4_ nanotubes/RGO. In addition, when the scavenger for h^+^ was added into the photocatalytic solution in Figure 7c, the degradation of acetaminophen was also depressed. It illustrates that h^+^ was involved as minor radical species in the photocatalytic process of BiVO_4_ nanosheets/RGO. As for the system based on BiVO_4_ nanoparticles/RGO, the reaction process was a little different. The degradation rate of acetaminophen in Figure 7a showed a certain decrease in the presence of formic acid, indicating h^+^ is the key reactive species responsible for the photodegradation over BiVO_4_ nanoparticles/RGO. According to the results mentioned above, we can preliminarily conclude that for BiVO_4_ nanoparticles/RGO photocatalytic process, h^+^ is the main radical species. In the BiVO_4_ nanotubes/RGO reaction system, •OH plays an important role, while in the BiVO_4_ nanosheets/RGO based reaction system, •OH and h^+^ as the major and minor radical species are all produced and participated in the acetaminophen degradation process.

Different from the BiVO_4_/RGO photocatalytic systems, pure BiVO_4_ samples take on the similar characteristics in Figure S8. For the pure BiVO_4_ nanoparticles, nanotubes and nanosheets, h^+^ is the main radical species participating the photocatalytic processes. 

**Figure 8:**
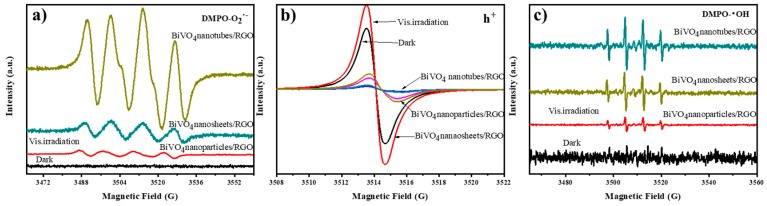
Electron spin resonance spectra of radical in BiVO_4_/RGO composites under visible light: (**a**) DMPO-O_2_^•−^, (**b**) h^+^ and (**c**) DMPO-•OH.

The involved ROS is further confirmed by ESR experiments under visible light. As is shown in Figure 8b, after the catalysts were exposed to visible light for 10 min, the characteristic peaks of h^+^ increased obviously for both BiVO_4_ nanoparticles/RGO and BiVO_4_ nanosheets/RGO than in dark condition, and the signals for BiVO_4_ nanotubes/RGO could nearly be ignored. Notably, the peak intensity of h^+^ referring to the BiVO_4_ nanosheets/RGO was much higher than that of BiVO_4_ nanoparticles/RGO. The result validated that the generation amount of h^+^ of this 2D-2D system was more than other nanocomposites. Moreover, from Figure 8c, obvious signals of Hydroxy-5, 5-dimethyl-1-pyrrolidinyloxy (DMPO-•OH) were also observed for BiVO_4_ nanotubes/RGO and BiVO_4_ nanosheets/RGO in the measurement under visible light irradiation, implying that •OH was produced in the above two reaction systems and took part in the photocatalytic process. It is noticeable that although stronger intensity DMPO- O_2_^•−^ and DMPO-•OH adducts were found in BiVO_4_ nanotubes/RGO system, BiVO_4_ nanosheets/RGO displayed higher photocatalytic efficiency for acetaminophen. This suggests that the photogenerated valence band hole of BiVO_4_ nanosheets/RGO can oxidize water to generate •OH and participated in the acetaminophen degradation process [42]. 

For comparison, the electron spin resonance spectra of the pure BiVO_4_ samples was demonstrated in Figure S9. Although the stronger signals of h^+^ and DMPO-O_2_•^−^ were observed with BiVO_4_ nanosheets, the photocatalytic degradation efficiency is similar to the BiVO_4_ nanotubes in Figure 5a and Table S1. It can be inferred that, although the photocatalytic activity of BiVO_4_ materials decreases dramatically after addition of formic acid, •OH dominants the acetaminophen degradation process. The inhibition of holes reduces the amount of reactive hydroxyl radicals, thereby reducing the photocatalytic activity of the system. Thus, this is to say, h^+^ as well as •OH was the main active species participating in the pure BiVO_4_ photocatalytic system.

**Scheme 1:**
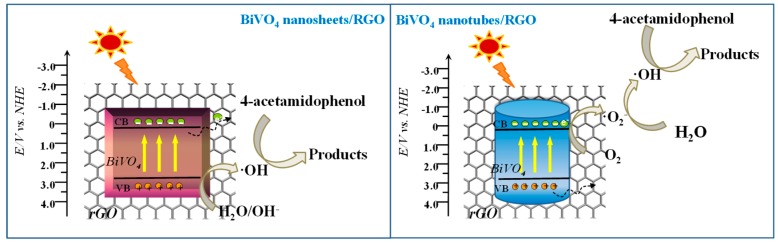
Schematic image of electron-hole separation mechanism for BiVO_4_/RGO.

It was reported that higher separation efficiency of electron-hole pairs plays a vital role in the photocatalytic degradation of pollutants [43,44]. According to the radical trapping experiment and ESR analysis, the reaction mechanism of 1D and 2D BiVO_4_/RGO heterojunctions for degrading organic pollutants was proposed (Scheme 1). It is assumed that 2D-2D heterojunction between BiVO_4_ nanosheets and graphene can facilitate the photogenerated electron transfer. That may promote the direct participation of holes in the photocatalytic process or the reaction with OH^−^ to generate •OH. 1D-2D heterojunction interfaces have the ability to yield more photo-generated electrons in the degradation process and promotes the oxygen molecules to generate O_2_^•−^ and then oxidized to get •OH. Besides, the stronger intensity of h^+^, O_2_^•−^, and •OH in 2D-2D system also demonstrates the intense interface facilitates more efficient charge separation and transfer. 

### 3.4. Photoinduced Electron Transfer Properties of BiVO_4_/RGO Composites 

In a photo-degradation process, the higher photocatalytic efficiency demands that the electron transfer is faster than the recombination [45]. The prolonged lifetime of the photogenerated electrons can be supported by Photoluminescence (PL) spectra in Figure S10. Under an excitation wavelength of 420 nm, the main emission peak of BiVO_4_ was detected at around 521 nm, owing to the recombination of electrons in the conduction band and holes in the valence band [46]. The introduction of graphene quenched the PL intensity of excited BiVO_4_ nanocomposites. The orders of the detected PL intensities were: BiVO_4_ nanoparticles > BiVO_4_ nanotubes > BiVO_4_ nanosheets > BiVO_4_ nanoparticles/RGO > BiVO_4_ nanotubes/RGO > BiVO_4_ nanosheets/RGO, which was in good accordance with the photocatalytic behaviors. Furthermore, the lower PL intensity of BiVO_4_ nanosheets/RGO suggests that the recombination of the photogenerated electron-hole pairs is efficiently inhibited by the two-dimensional heterjunction interface and the charge carriers separation rate is promoted. 

**Figure 9:**
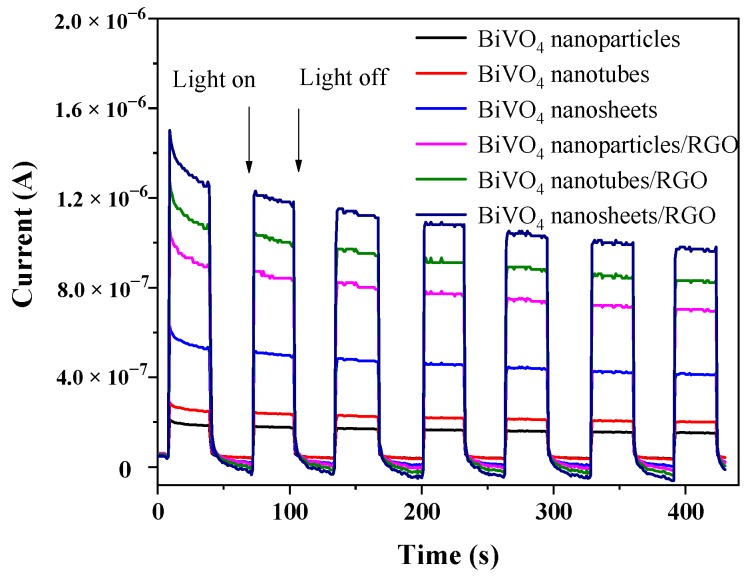
Photocurrent responses of the prepared nanocomposites in 0.5 M Na_2_SO_4_ solution during the repeated on-off cycles under visible light irradiation.

The enhanced charge transfer rate of BiVO_4_ nanosheets/RGO was further demonstrated by the transient photocurrent responses. As displayed in Figure 9, the photocurrent density of the BiVO_4_/RGO composites is much higher than that of the pure BiVO_4_ samples, especially for BiVO_4_ nanosheets/RGO. That is in good accordance with the result of photocatalytic performances. It is indicated that the enhanced photocurrent response of the BiVO_4_ nanosheets/RGO represents higher efficiency of charge separation and lower recombination rate in 2D-2D heterojunction interface [47]. 

On the basis of the above results, the charge transfer mechanism could be proposed as follows. Due to the intimate contact of 2D-2D interface, the favorable transfer of electrons from BiVO_4_ nanosheets to graphene can reduce the recombination of electron–hole pairs. The enhanced generation of ROS, especially h^+^ and •OH, accelerate the photocatalytic degradation process of acetaminophen.

## 4. Conclusions

In summary, the BiVO_4_ nanoparticles/RGO, BiVO_4_ nanotubes/RGO and BiVO_4_ nanosheets/RGO hybrids were prepared and the photocatalytic performance was evaluated. The morphology, chemical structures and photocatalytic performance of the as-prepared samples are studied through a series of characterization. Compared to 0D/2D and 1D/2D nanocomposites, 2D/2D BiVO_4_ nanosheets/RGO with two-dimensional interface exhibits higher photoactivity. That can be attributed to a stronger electronic and physical coupling effect between BiVO_4_ nanosheets and graphene nanosheets, which allows for the prolonged lifetime and effective separation of electrons and holes. The 2D-2D heterojunction interface opens a new window for exploiting a visible light photocatalytic system with well-defined nanohybrids to purify the polluted environment.

## Supplementary Materials

The following are available online at https://www.mdpi.com/2079-4991/9/6/907/s1, the details of preparation of BiVO_4_ nanoparticles and BiVO_4_ nanoparticles/RGO, Figure S1: Atomic force microcopy images of the 2D BiVO_4_ nanosheets, Figure S2: Thermo gravimetric analysis (TGA) of the BiVO_4_/RGO composites, Figure S3: XRD patterns of the prepared graphene oxide (GO), Figure S4: Raman spectra of BiVO_4_ and BiVO_4_/RGO composites, Figure S5: HPLC chromatograms of acetaminophen standard sample, Figure S6: The adsorptive performance of acetaminophen over the BiVO_4_ and BiVO_4_/RGO composites without visible light irradiation, Table S1. The pseudo-first order kinetic equation and the rate constant (k) of BiVO4 and BiVO4/RGO composites, Figure S7: Photocatalytic degradation of RhB over photocatalysts under different pH conditions: (a) BiVO_4_ nanosheet/RGO; (b) BiVO_4_ nanotube/RGO;(c) BiVO_4_ nanoparticle/RGO, Figure S8: Free radical inhibition experiment of BiVO_4_ samples: (a) BiVO_4_ nanoparticles; (b) BiVO_4_ nanotubes; (c) BiVO_4_ nanosheets, Figure S9: Electron spin resonance spectra of radical in BiVO_4_ samples under visible light: (a) DMPO-O_2_•^−^, (b) h^+^ and (c) DMPO-•OH, Figure S10: PL spectra of BiVO_4_ and BiVO_4_/RGO samples.
