# Supplementary material for: Engineering the Dimensional Interface of BiVO4-2D Reduced Graphene Oxide (RGO) Nanocomposite for Enhanced Visible Light Photocatalytic Performance
Source: Nanomaterials (Basel). 2019 Jun 10;9(6):907. doi: 10.3390/nano9060907 (PMC6630799; doi:10.3390/nano9060907)
Supplement: Supplementary file 1 [file nanomaterials-09-00907-s001.pdf]

Supporting Information for Nanomaterials

Engineering the Dimensional Interface of $BiVO_4$-2D Reduced Graphene Oxide (RGO) Nanocomposite for Enhanced Visible Light Photocatalytic Performance

Jing Sun [1,*,†], Chunxiao Wang [1,†], Tingting Shen [1], Hongchen Song [1], Danqi Li [1], Rusong Zhao [2] and Xikui Wang [3,*]

[1] School of Environmental Science and Engineering, Qilu University of Technology (Shandong Academy of Sciences), Ji'nan 250353, China; wangyiwangchunxiao@163.com (C.W.); stthunanyt@163.com (T.S.); shc01261005lx@gmail.com (H.S.); autantenporte@163.com (D.L.)

[2] Key Laboratory for Applied Technology of Sophisticated Analytical Instruments of Shandong Province, Analysis and Test Center, Qilu University of Technology (Shandong Academy of Sciences), Ji'nan 250014, China; zhaors1976@126.com

[3] College of Environmental Science and Engineering, Shandong Agriculture and Engineering University, Ji'nan, 251100, China

* Correspondence: jingsunok@163.com (J.S.); xk_wang@qlu.edu.cn (X.W.)

† These authors contributed equally to this work.

(7 Pages, 10 Figures)

Preparation of $BiVO_4$ nanoparticles. 2.91 g Bi $(NO_3)_3$ $5H_2O$ and 0.7204 g NH_4VO_3 were added into a beaker containing 30 mL 1 M HNO_3 solution. After stirring for a few minutes, 3 g $(NH_2)_2CO$ was gradually added into the above solution and then placed in a water bath at a temperature of 80 °C for 24 h. The solution was centrifuged to obtain the yellow sediment and washed with deionized water. The $BiVO_4$ nanoparticles were dried at 60 °C in an oven and then calcined at 400 °C for 1 h.

Preparation of $BiVO_4$ nanoparticles /RGO Composites. The $BiVO_4$ nanoparticles and GO solution (1g L^{-1}) were dissolved into 50 mL ultrapure water and stirred to ensure complete mixing. Then, the obtained solution was transferred to Teflon-lined autoclave, and maintained at 160 °C for 6 h in an oven. The above product was washed with alcohol and ultrapure water several times, centrifuged, dried at 60 °C in an oven.

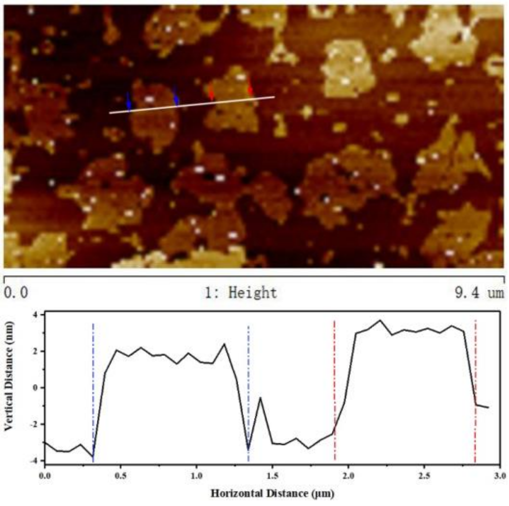

Figure S1. Atomic force microcopy images of the 2D $BiVO_4$ nanosheets.

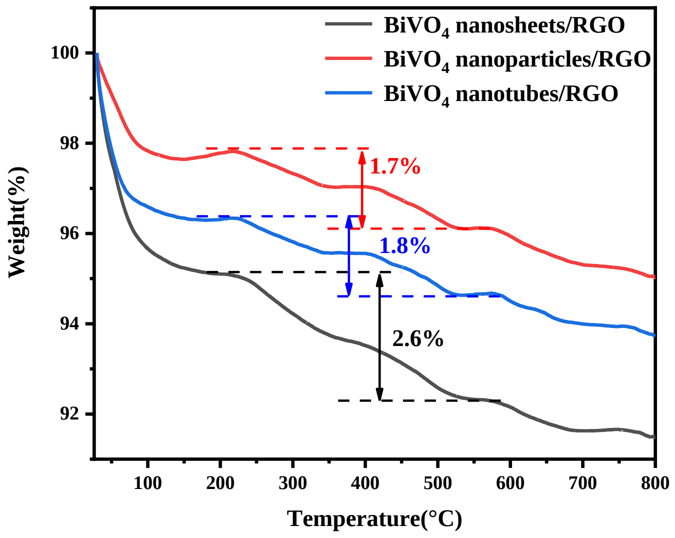

Figure S2. Thermo gravimetric analysis (TGA) of the $BiVO_4$/RGO composites.

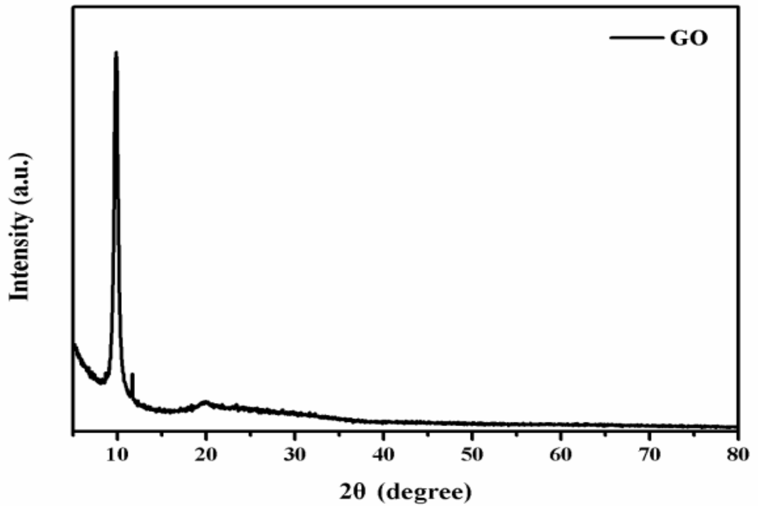

Figure S3. XRD patterns of the prepared graphene oxide (GO).

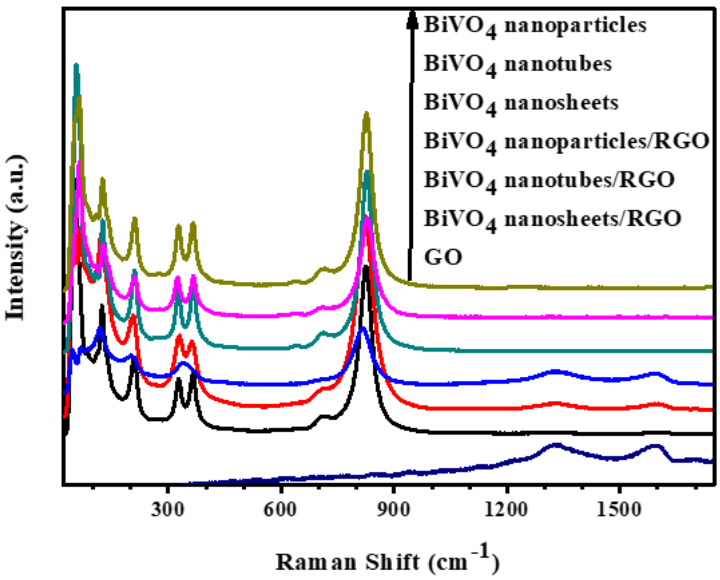

Figure S4. Raman spectra of $BiVO_4$ and $BiVO_4$/RGO composites.

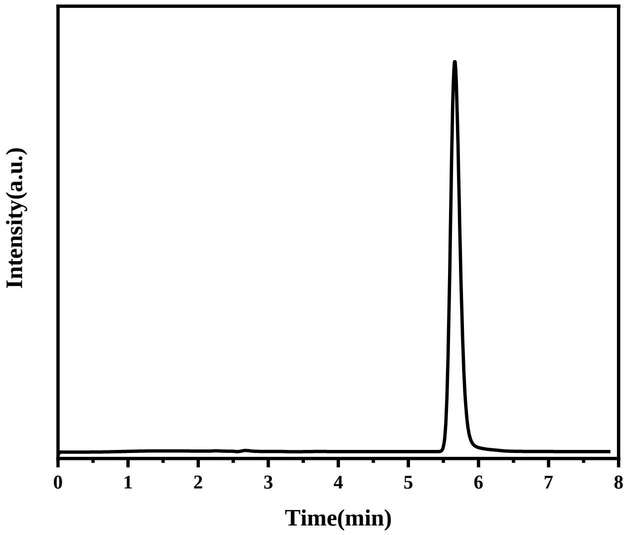

Figure S5. HPLC chromatograms of acetaminophen standard sample.

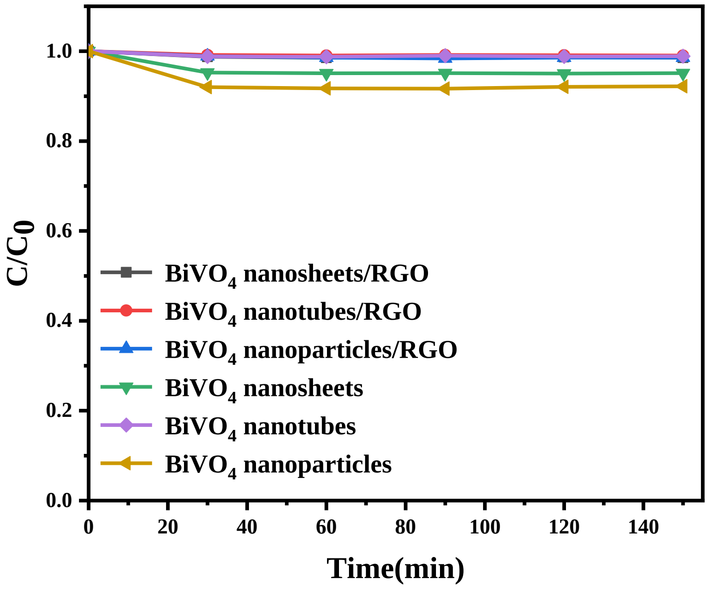

Figure S6. The adsorptive performance of acetaminophen over the $BiVO_4$ and $BiVO_4$/RGO composites without visible light irradiation.

Table S1. The pseudo-first order kinetic equation and the rate constant (*k*) of $BiVO_4$ and $BiVO_4$/RGO composites.

Samples	First-order kinetic equation	*k* (min^{-1})	R^2
$BiVO_4$ nanosheets/RGO	$-\ln(C/C_0) = 0.0141x - 0.0472$	0.0141	0.9976
$BiVO_4$ nanotubes/RGO	$-\ln(C/C_0) = 0.0122x - 0.0684$	0.0121	0.9963
$BiVO_4$ nanoparticles/RGO	$-\ln(C/C_0) = 0.0107x - 0.0292$	0.0107	0.9990
$BiVO_4$ nanosheets	$-\ln(C/C_0) = 0.008x - 0.0142$	0.0080	0.9986
$BiVO_4$ nanotubes	$-\ln(C/C_0) = 0.0084x - 0.0737$	0.0084	0.9915
$BiVO_4$ nanoparticles	$-\ln(C/C_0) = 0.0077x - 0.0889$	0.0077	0.9924

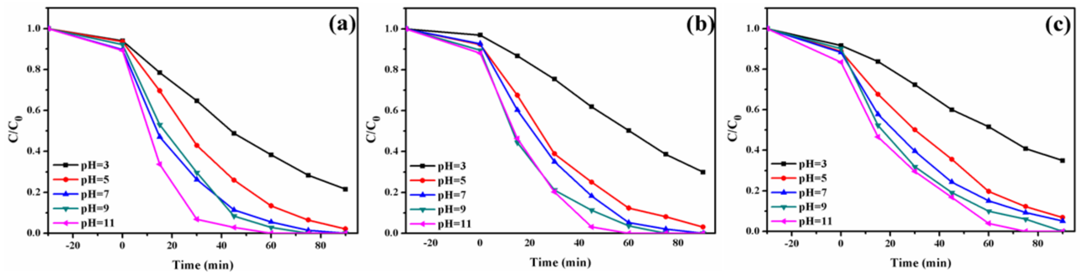

Figure S7. Photocatalytic degradation of RhB over photocatalysts under different pH conditions:(a) $BiVO_4$ nanosheet /RGO; (b) $BiVO_4$ nanotube /RGO;(c) $BiVO_4$ nanoparticle /RGO.

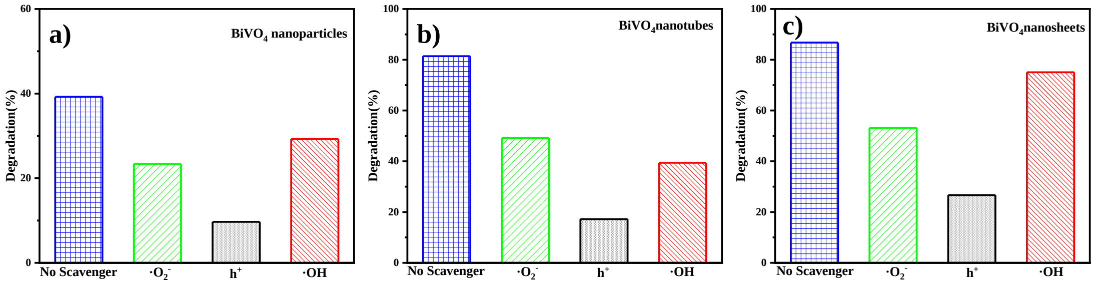

Figure S8. Free radical inhibition experiment of $BiVO_4$ samples: (a) $BiVO_4$ nanoparticles; (b) $BiVO_4$ nanotubes; (c) $BiVO_4$ nanosheets.

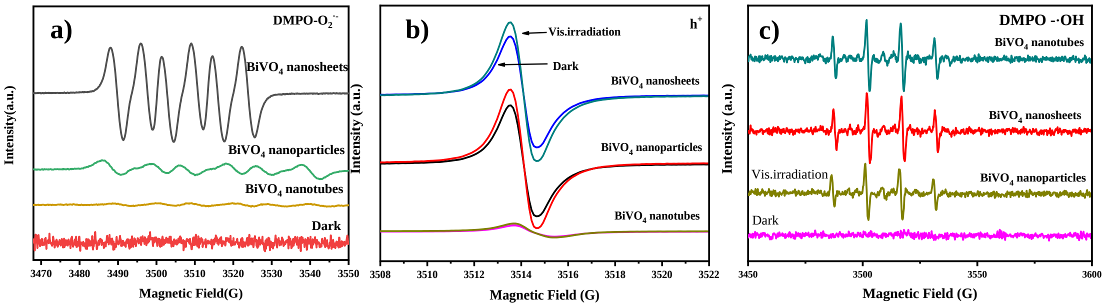

Figure S9. Electron spin resonance spectra of radical in $BiVO_4$ samples under visible light: (a) DMPO-O_2•⁻, (b) h^+ and (c) DMPO-•OH.

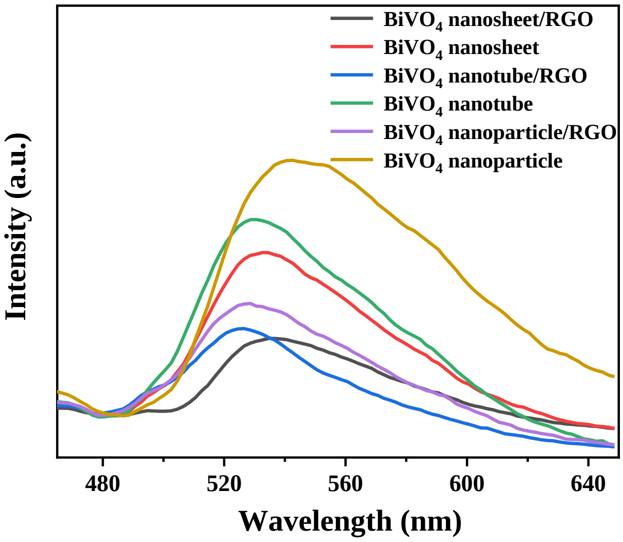

Figure S10. PL spectra of $BiVO_4$ and $BiVO_4$/RGO samples.
